# Race and Socioeconomic Status: Interlinked Drivers of Sleep Health Disparities

**DOI:** 10.1089/heq.2023.0012

**Published:** 2023-05-26

**Authors:** Oragun Rojanapairat, Abigail Beggs, Michelle Zeidler, Bharati Prasad

**Affiliations:** ^1^ Department of Medicine, Cedars-Sinai Medical Center, Los Angeles, California, USA.; ^2^ Department of Medicine, Veterans Affairs Greater Los Angeles Healthcare, Los Angeles, California, USA.; ^3^ Department of Medicine, Jesse Brown VA Medical Center and the University of Illinois at Chicago, Chicago, Illinois, USA.

**Keywords:** sleep apnea, PAP, race, socioeconomic status, veterans, insomnia, sleep deprivation, racial minority, minority health

## Abstract

The effect of race and socioeconomic status on sleep disorders has significant effects on the availability of healthcare and health outcomes. This paper examines how race and SES contribute to sleep health disparities, emphasizing the importance of understanding their impact on sleep disorders and treatment particularly in minority populations and veterans.

## Introduction

By 2050 more than half of the population in the United States (U.S.) will be identified as minority. In a survey by the Pew Research Center in 2019, Americans expected more difficult access to health care, a wider income gap, a larger lower socioeconomic class, and a decline in the standard of living for the average American family.^1,2^

The sources of health care disparities include geographic location, lack of access to adequate health coverage and providers, communication difficulties between patient and provider, cultural barriers (e.g., religion), provider stereotyping, race, ethnicity, and socioeconomic status (SES).^3^ Historically racial and ethnic minorities have received lower quality care than majority populations when controlling for SES with factors such as insurance status, education, and income.

In a study by the Kaiser Family Foundation in 2021, 21.7% of Native Americans, 20% of Hispanics, 12.7% of Pacific Islanders, 11.4% of African Americans (AA), 7.4% of Asians, and 7.8% of Whites lacked health insurance.^4^ The lack of health insurance reflects a lack of access to routine health care, possibly limited health literacy, and a lack of financial stability or lower SES. Since 2019, given the loss of many low wage jobs due to pandemic-related closures, these disparities have become more stark, and vulnerable groups have had significant setbacks in usage of routine and emergency health care.

Good quality sleep is important to every person's health. Sleep deprivation and common sleep disorders have been identified as a major unmet public health problem in the United States.^5^ There has been significant growth of knowledge in the understanding and treatment of sleep disorders, but access to and broad implementation of these advances haves not been equitable for all individuals. Here we review some of the common sleep disorders and disparities linked to both race and SES and how those disparities also manifest within veterans.

## Methods

A literature search using PubMed and Google Scholar for articles included the following terms: sleep apnea, race, socioeconomic status, veterans, insomnia, sleep deprivation, racial minority, and minority health.

### Sleep duration and sleep deprivation

In the United States, ∼30–40% of adults and 40–70% of adolescents report sleep deficiencies with the highest prevalence seen in populations with racial minorities and low SES.^6,7^ Short sleep duration (fewer than 7 h of sleep) is associated with increased risk of developing comorbid cardiovascular conditions (i.e., obesity, diabetes, hypertension, coronary artery disease [CAD], and stroke), as well as mental health disorders and increased mortality. Suboptimal sleep duration is associated with lapses of attention, slower working memory, and depressed mood, leading to poor performance and increased likelihood of motor vehicle accidents.^8–11^

Several studies have explored sleep differences between racial and ethnic groups in the United States. When using self-reported sleep measures, AA reported lower sleep duration than Whites using sleep diaries.^12^ Surveys found AA and Hispanics were more likely to report short sleep duration, and AA were more likely to report long sleep duration.^13–16^ These findings were consistent when controlling for SES.^17^ Studies using polysomnography, where sleep time is accurately measured, reported AA were more likely to have lower sleep efficiency and less slow-wave sleep than Whites.^18^

The Chicago Area Sleep Study (CASS) and Coronary Artery Risk Development in Young Adults Study (CARDIA) used actigraphy to quantify sleep duration. Again, it was shown that AA and Hispanics had shorter sleep duration.^18,19^ Education was examined within each race in the CASS study and found to be a moderator of sleep continuity in AA and Hispanics, and daytime sleepiness in AA. Neither study examined the effect of race on sleep outcomes in multivariable models controlling for SES.

Veterans represent another at-risk population and have a high incidence of sleep disorders. They are at risk for post-traumatic stress disorder (PTSD), depression, panic disorders (PDs), and substance abuse.^20–22^ PD was significant when controlling for “minority status” (not defined) in one study.^22^ Low SES was associated with short sleep duration, but race was not significant.^23^ The prevalence of poor sleep has been reported as high as 89%, of which 20% reported sleeping <4.5 h a night. In addition, sleep problems were more severe in veterans with low SES, greater combat exposure, and comorbid psychiatric disturbances.^24^

Few studies of sleep disorders in veterans assess race beyond the distinction of White versus non-White. The findings of these studies are not consistent when reviewing secondary end-points but suggest that in addition to the increased risk veterans face, there is additional risk of short sleep in both racial minorities and low SES.^22–24^

### Obstructive sleep apnea

Obstructive sleep apnea (OSA) is a highly prevalent sleep disorder. The diagnosis of OSA is associated with an increased risk of cardiovascular disease, metabolic syndrome, chronic kidney disease (CKD), and death. A retrospective cohort study of 3 million U.S. veterans with near normal kidney function evaluated the outcomes of 21,000 patients diagnosed with incident OSA. OSA was associated with higher mortality (86% untreated vs. 35% treated), higher risk of CAD, higher risk of stroke, higher risk of CKD, and faster decline in kidney function, all with hazard ratios of 2 or greater.

The analyses were controlled for race and income, however, there was no comment on individual effects of either. Interestingly, minorities (AA, Hispanics, and other non-Whites) were less likely to have incident OSA than White people in this study.^25^ In contrast, multiple other studies have found that racial minorities are more likely to be diagnosed with OSA than White people.^26–28^ Residents of neighborhoods with a low walkability score (surrogate for low SES) are at increased risk for OSA and greater severity of OSA, with a weaker but similar association with AA and Hispanic race.^29^ OSA and daytime sleepiness appear to be more severe in AA than in White people.^30,31^ AA are less likely to self-refer to sleep specialists than White people, and when referred there is lower (38%) follow through.^32,33^

Positive airway pressure (PAP) is the first-line and highly efficacious therapy for OSA. Treatment with PAP has been previously demonstrated to reduce daytime sleepiness, improve blood pressure, lower risk of motor vehicle crashes, and improve subjective quality of life. Common barriers to PAP adherence in all groups include anxiety, claustrophobia, and comorbid insomnia.^34^ However, when treatment with PAP is recommended, AA, Hispanic, and residents of low SES neighborhoods are less likely to accept therapy.^35–38^ Higher average systolic blood pressure (SBP) is seen in low SES groups not on PAP therapy, but there is a greater reduction in SBP with increasing use of PAP in this group. Race does not have an independent effect on blood pressure or blood pressure response with PAP therapy when adjusted for SES.^39^

In veterans, OSA is the most common sleep disorder (47%).^40^ There are limited data on veterans with sleep disorders and low SES or racial minorities. At 30 days, PAP adherence among veterans (controlled for SES) was 50% overall, but 42% in AA versus 53% in non-AA. Only 8% of the participants in this study had low SES.^41^ PTSD is a common comorbidity among veterans and can impact treatment of all concurrent health conditions. PAP adherence is reduced with comorbid PTSD, particularly when nightmares are present.^42^ In a prospective study of veterans with PTSD and OSA, PAP usage resulted in improvements in both subjective sleepiness, sleep quality, quality of life, and PTSD symptoms.^43^ Data on the impact of SES on PAP adherence in these groups are limited.

### Insomnia

Insomnia is a common sleep complaint. Insomnia is defined as difficulty initiating sleep and maintaining sleep that leads to an impairment in daytime functioning. Patients may manifest symptoms such as fatigue, daytime sleepiness, impaired concentration, mood disturbances, impaired work performance, and/or poor quality of life. Insomnia with short sleep duration increases the risk of heart disease, hypertension, diabetes, and death.^44–48^ Higher prevalence of insomnia is seen in women, elderly population, racial minorities, lower SES, and poor health or quality of life.^49^ Insomnia is a substantial burden for the U.S. health care system and is estimated to cost >$100 billion annually.^50,51^

Insomnia is the second most common sleep disorder in veterans,^40,52^ with an incidence that varies between 30% and 57%, versus 30% in the general population. Minority veterans have slightly higher rates of insomnia than Whites; lower SES is also associated with increased rates of insomnia.^53,54^ Women veterans have similar rates of insomnia to race and SES-matched male veterans.^55^

PTSD, traumatic brain injury, and chronic pain are known to contribute to the development of insomnia in this population.^53^ Cognitive behavioral therapy for insomnia (CBT-I) is the first-line therapy for insomnia. Nationwide access is extremely limited, in even resource-rich settings, but is perhaps most available within the VA health care system. Little research has been done on the effectiveness of CBT-I in minorities or low SES groups.

## Conclusion

There are significant differences related to race and SES in prevalence, access to care, and the use of treatment in sleep deficiency and common sleep disorders. The American College of Physicians recommends affordable access to health care to eliminate racial and ethnic disparities.^56^ Physicians and allied health care providers need to be aware of differences and provide appropriate care. Future research on disparities must include minorities and low SES groups to rigorously examine the effect of sleep disorders and their treatment in these groups. This research should be used to inform public policy and clinical practice guidelines to mitigate sleep health disparities.

## Abbreviations Used

AA: African Americans
CAD: coronary artery disease
CARDIA: Coronary Artery Risk Development in Young Adults Study
CASS: Chicago Area Sleep Study
CBT-I: cognitive behavioral therapy for insomnia
CKD: chronic kidney disease
OSA: obstructive sleep apnea
PAP: positive airway pressure
PD: panic disorders
PTSD: post-traumatic stress disorder
SBP: systolic blood pressure
SES: socioeconomic status
